# Platelet Pathogen Reduction Technology—Should We Stay or Should We Go…?

**DOI:** 10.3390/jcm13185359

**Published:** 2024-09-10

**Authors:** Andrea Piccin, Allameddine Allameddine, Gilbert Spizzo, Katrina M. Lappin, Daniele Prati

**Affiliations:** 1 Northern Ireland Blood Transfusion Service (NIBTS), Belfast BT9 7TS, UK; allameddine.allameddine@nibts.hscni.net; 2 Department of Internal Medicine V, Medical University of Innsbruck, 6020 Innsbruck, Austria; 3 Department of Industrial Engineering, University of Trento, 38122 Trento, Italy; 4 Department of Oncology, Brixen Hospital, 39042 Bolzano, Italy; gilbert.spizzo@gmail.com; 5 Patrick G Johnston Centre for Cancer Research, Queen’s University Belfast, Belfast BT7 1NN, UK; k.lappin@qub.ac.uk; 6 Servizio Trasfusionale, Ospedale Ca’ Granda, 20122 Milano, Italy; daniele.prati@policlinico.mi.it

**Keywords:** platelets, INTERCEPT^®^, THERAFLEX^®^, MIRASOL^®^, amotosalen, riboflavin, bacterial contaminations, irradiation, TA-GvHD

## Abstract

The recent COVID-19 pandemic has significantly challenged blood transfusion services (BTS) for providing blood products and for keeping blood supplies available. The possibility that a similar pandemic event may occur again has induced researchers and transfusionists to investigate the adoption of new tools to prevent and reduce these risks. Similarly, increased donor travelling and globalization, with consequent donor deferral and donor pool reduction, have contributed to raising awareness on this topic. Although recent studies have validated the use of pathogen reduction technology (PRT) for the control of transfusion-transmitted infections (TTI) this method is not a standard of care despite increasing adoption. We present a critical commentary on the role of PRT for platelets and on associated problems for blood transfusion services (BTS). The balance of the cost effectiveness of adopting PRT is also discussed.

## 1. Introduction

### Overview of Platelet Transfusions and Transfusion-Transmitted Infections Risks

The risk of transfusion-transmitted infections (TTI) in resource-rich countries has been significantly reduced due to the continuous implementation of donor selection policies, enhanced serological testing, the introduction of nucleic acid testing (NAT), and other crucial measures such as precise skin disinfection and the use of diversion bags during blood collection. Current risks vary according to local epidemiology but are deemed acceptable by most national transfusion systems. According to recent data from the UK and the US, the risks are as follows: HBV 1 in 3 million donations, HIV 1 in 5 million donations, and HCV 1 in 1 million donations [1].

Nevertheless, the possibility of acquiring infections from emerging pathogens (e.g., the West Nile virus and Dengue virus), as well as from bacteria and protozoa, remains a concern in transfusion medicine. Bacterial contamination of stored blood products, particularly platelets (PLTs), is still a threat for the safety of blood supply. PLT bags are kept on agitators at room temperature (20–24 °C) to preserve PLT quality and function, but this storage condition can facilitate bacterial growth, leading to transfusion reactions and sepsis. This risk is not negligible; a meta-analysis estimates it to be approximately 1 in 1900 for buffy coat (BC) PLTs and 1 in 4348 for apheresis-collected PLTs [2]. Furthermore, one-quarter of contaminated PLT bags result in sepsis [3].

Blood transfusion services may choose to mitigate this risk using bacterial screening approaches. The BacT/ALERT© system, is an efficient tool that promptly detects bacterial growth by measuring CO_2_ production in samples from PLT bags. Samples are incubated in dedicated culture bottles, and any increase in CO_2_ triggers an alarm, prompting the operator to remove the implicated unit from the blood product inventory. The unit remains quarantined until further investigation. However, BacT/ALERT© cannot always detect bacteria such as *Staphylococcus epidermidis*, *Staphylococcus aureus*, and other species [4]. Additionally, BacT/ALERT© has a false positivity rate of 0.2–3.2%, leading to unnecessary product discards [5].

In addition, a further reduction in the possibility of acquiring TTIs is crucial to ensure patient safety and maintain product supply. Globalization and international travel have increased TTI risks, particularly from zoonoses. This was highlighted at the onset of the COVID-19 pandemic, with concerns about potential blood transmission. Another challenge for BTS is emerging pathogens. When a new pathogen, such as the Chikungunya or Zika virus, emerges, specific measures like donor deferral, screening, and recall are adopted, significantly impacting donor pools. Implementing these measures requires substantial time, staff, and resources, and their efficacy in reducing TTI risk is only partial [6]. To prevent TTIs and ensure adequate blood stocks, BTS often import blood products from other regions or countries, leading to service disruptions and high costs.

Evaluating and implementing new pathogen reduction technology (PRT) is thus essential. A recent systematic review by Angel Gimenez-Richarte and colleagues demonstrated that UV-based PRT is particularly effective against arboviruses, significantly reducing the viral load of West Nile virus, Chikungunya virus, Dengue virus, and Zika virus [7]. The efficacy of UV-based PRT varies with the inactivation method used. Importantly, PRT does not neutralize donor antibodies (e.g., convalescent plasma), allowing the recipients of treated blood products to benefit from donor antibodies against new infectious agents.

Recently, the European Directorate for the Quality of Medicines (EDQM) suggested implementing PRT as a part of blood processing in its Blood Supply Contingency and Emergency Plan (B-SCEP) (https://freepub.edqm.eu/publications/PUBSD-174/detail, Accessed on 31 August 2024). Implementing PRT will help harmonize TTI risks across countries. The new PRT types available use UVA, UVB, and UVC light and may include special compounds that crosslink or associate with DNA/RNA after UVA irradiation (e.g., INTERCEPT^®^ and MIRASOL^®^) or UVC light only (THERAFLEX^®^). This review aims to revisit existing knowledge on the efficacy, practicality, and challenges of using UV light-mediated PRT systems for PLTs.

## 2. Understanding Pathogen Reduction Technology (PRT)

### 2.1. Explanation and Evolution of PRT (Short Analysis of the Three Main Methods)

The need to ensure better blood safety and to prevent TTI became urgent following the blood scandals of the 1980s and early 1990s. In the attempt to reduce plasma contamination, treatment with solvent detergent (SD) was adopted for blood-derived products (not for PLT). This is an excellent first example of PRT for blood-derived products. SD is very effective against lipid-enveloped viruses (also against protozoa), but not against unenveloped viruses such as HAV. Also, SD badly affects alpha-2 antitrypsin and Protein-S (an important naturally occurring anticoagulant) [8]. Another drug, similarly used as a PRT agent, is methylene blue (MB). In 2003, to reduce the risk of transmission of variant Jacob Creutzfeldt Disease (vCJD) for the population born post-1996, fresh-frozen plasma (FFP) was outsourced and imported from the USA (lower vCJD geographical risk). At the time, plasma was also treated with MB to ensure that the attempt towards the reduction in vCJD risk would have not caused an increase in other blood-borne viruses. MB is associated with allergic reactions and hypotension, currently its use is not recommended. In Belgium and in some other countries (e.g., Poland and Spain) it is still used as an accepted PRT method. Recently, UV-based PRT systems became available, and this is the topic of this review.

### 2.2. Short Analysis of the Three Main Methods

The existing pathogen reduction (PR) technology systems for platelets based on UV light are the following: (i) INTERCEPT^®^ PR system, (ii) MIRASOL^®^ PR system, (iii) THERAFLEX^®^ UVC PR system [9].

**Figure 1:**
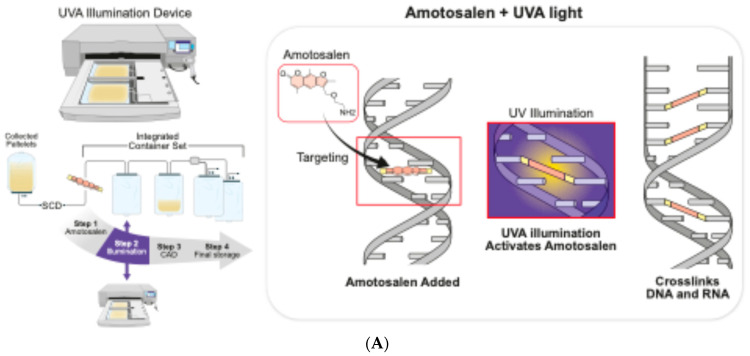

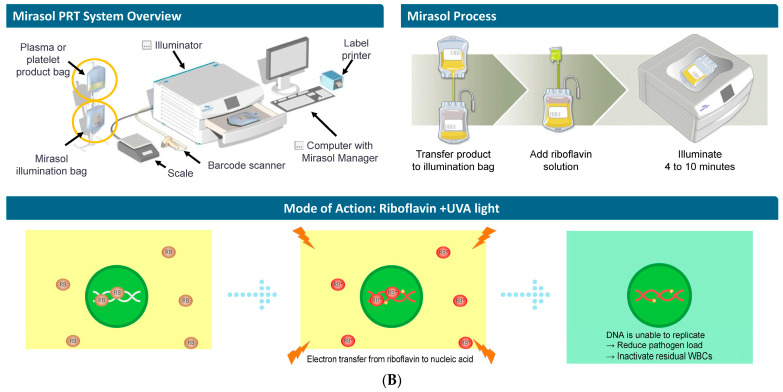
(**A**) The first cartoon shows the mechanism of action of the INTERCEPT^®^ PR system. The inactivation process is based on a direct crosslinking of amotosalen with the DNA chain, following exposure to UVA light (step 2). After the UVA illumination step, the CAD device is used to remove any amotosalen in excess to reduce toxicity. (**B**) The mechanism of action of MIRASOL^®^ PR system. Riboflavin acts as a photosensitizer that is associated with nucleic acids and mediates an oxygen-independent electron transfer process leading to the modification of DNA/RNA upon exposure to UV light. This system utilizes riboflavin (vitamin B2) which is not toxic, therefore a CAD device is not needed.

The exact mechanism of action of these three methods are illustrated in Figure 1. Key characteristics are also reported in Table 1.

We will now analyze all three methods in depth.

I. INTERCEPT^®^ PR system

This PRT method uses amotosalen as the inactivation agent. Amotosalen is a synthetized photoactive psoralen compound. PLTs are resuspended in approximately 35% plasma and 65% PAS, or in 100% plasma. Amotosalen undergoes photoactivation with UVA light at 320–400 nm. A total dose of 3 joules/cm^2^ is delivered, causing amotosalen to crosslink nucleic acids (DNA or RNA). Over time, this binding becomes permanent, impairing the replication of bacteria and other microorganisms. The residual amotosalen is subsequently removed using a dedicated compound adsorbtion device (CAD), which results in a certain degree of platelet loss (between 7 and 10%) (Figure 1A). For these reasons, Gathof BS et al. recommend increasing the lower limit of PLT units to 2.5 × 10^11^ [10]. However, the recent EDQM guidelines have suggested a lower limit of 2.0 × 10^11^ [11].

The efficacy of the INTERCEPT^®^ PR system has been evaluated in several randomized clinical trials. McCullough reported on a randomized study in bleeding patients (SPRINT trial) with WHO grade 2 degrees (mild blood loss, clinically significant). Patients in the study group were transfused with PLT treated with the INTERCEPT^®^ PR system, while patients in the control group received standard PLT [12]. This study did not show a difference in bleeding events; however, patients transfused with PRT PLT required more products. It also emerged that PRT units had a lower concentration of PLT (<3.0 × 10^11^). In the same study, post-transfusion CCI at 1 h and at 24 h were lower in the arm photochemically treated (PCT) with PRT-PLT. The transfusion interval was shorter, leading overall to a higher prescription of PLT. Nevertheless, no differences in clinical bleeding were seen. Additionally, fewer transfusion reactions were observed when PRT PLT was administered (3.0% PCT versus 4.4% control; *p* = 0.02) [12].

Another randomized study (PRT-treated PLT unit versus standard PLT unit) on the INTERCEPT^®^ PR system, carried out in over 100 patients with thrombocytopenia (euroSPRITE), showed no statistically significant difference in CCI at 1 h and at 24 h of follow-up. Patients treated with PRT units had slightly lower platelet counts. Hemorrhagic and thrombotic events after transfusion were similar in both groups. However, in 2010, worrisome results were highlighted by the Dutch–Belgian HOVON cooperative group. In a randomized study, Kerkhoffs JLH et al. showed that the use of the INTERCEPT^®^ PR system was associated with decreased CCI and with grade ≥ 2 bleeding. These findings were not confirmed by randomized controlled trials such as the SPRINT trial and the EFFIPAP trial, where the primary endpoint was WHO grade-2 bleeding [13]. However, in the EFFIPAP trial, the results differed depending on the control arm used (PLT in PAS or PLT in plasma). In the HOVON study, amotosalen was added to platelets suspended in approximately 35% plasma/65% PASIII (study group) and compared with PLT resuspended in plasma only and PASIII only (control groups) [14]. This may have in part contributed to the overall negative results. Moreover, a recent review of PRT types and their efficacy showed that INTERCEPT^®^ PR system is certainly more efficacious against arboviruses compared to MIRASOL^®^ PR system [7]. This is also confirmed by a better Log Reduction Factor (LRF) of 4 or more (>4 log10) with the INTERCEPT^®^ PR system. Similarly, the INTERCEPT^®^ PR system inactivates leukocytes, and its implementation could also make irradiation redundant [15]. Moreover, the possibility of a 7 day platelet storage has also been shown with the INTERCEPT^®^ PR system [16].

II. MIRASOL^®^ PR system

This PRT method utilizes riboflavin (vitamin B2), a photoreactive compound that is non-toxic (Figure 1B). Therefore, no further manipulation of the blood components is required after the illumination step, resulting in minimal loss of platelets during the inactivation treatment.

The compound is released in platelets resuspended either in plasma or in plasma with platelet additive solution (PAS). This is followed by photoactivation of riboflavin using UV-B light with a total energy power of 6.2 Joules/cm^2^. A compound adsorption device (CAD) is not required because riboflavin is non-toxic. This method is highly effective against enveloped viruses, such as influenza virus, cytomegalovirus (CMV), HIV, respiratory syncytial virus (RSV), and human coronaviruses.

One of the most notable results achieved by the MIRASOL^®^ PR system was demonstrated by Allain JP and colleagues in the AIMS study conducted in Ghana where the risk of blood parasitemia by malaria is >50% [17,18]. In this study, whole blood treated with the MIRASOL^®^ PR system showed a reduction in the incidence of transfusion-transmitted malaria (*p* = 0.039) [18]. Furthermore, the MIRASOL^®^ PR system has been reported to have moderate inactivation of enveloped viruses such as HEV [19,20].

Randomized studies have shown a 25% decrease in corrected count increment (CCI) when platelets inactivated with riboflavin-PRT were infused. This finding was confirmed in another study at 1 h after transfusion. However, no major bleeding or other relevant side effects were observed, although a larger requirement of platelets in some patient categories was reported [21]. A recent study (miPLATE trial) did not show an inferiority of the MIRASOL^®^ PR system compared to conventional platelets when the endpoint was calculated in days ≥ Grade 2 bleeding [22].

Additionally, the platelet shelf life was extended to 7 days without any major side effects, except for a higher rate of lactate increase and glucose depletion starting from day 5. The platelet loss with the MIRASOL^®^ PR system was lower than previously reported with the INTERCEPT^®^ PR system, although a loss of the swirling effect and increased apoptosis (assessed by Annexin) were also reported [23]. Furthermore, the MIRASOL^®^ PR system inactivates leukocytes. This could theoretically suggest that leukoreduction/filtration may not be needed, but Norris P and colleagues have found contradictory results [24].

The main side effect noted with the MIRASOL^®^ PR system is ATP consumption, which suggests that glycolytic pathways are activated [25]. A randomized study (MERIT trial) from Uganda University is currently underway to clarify the potential benefits of the MIRASOL^®^ PR system for transfusion-transmitted infection (TTI) prevention [17].

III. THERAFLEX^®^ UVC PR SYSTEM

The THERAFLEX^®^ UVC PR system is based on the use of UVC (wavelength 200–280 nm) [26,27]. This system is highly promising against high concentrations of various bacterial species [28]. However, it is not very effective against HIV inactivation. The strongest efficacy (4 or more log steps) was demonstrated against the vesicular stomatitis virus, porcine parvovirus (a model virus for parvovirus B19), encephalomyocarditis virus (a model virus for hepatitis A), and Sindbis virus (a model virus for hepatitis C). Other viruses, such as the Pseudorabies virus (a model virus for hepatitis B) and West Nile virus, were less effectively inactivated, with reduction factors of approximately 2–3 and 3.5–4 log steps, respectively [27]. Eickmann M and colleagues conducted an in vitro study demonstrating that THERAFLEX^®^ UVC PR system can effectively reduce the infectivity of severe acute respiratory syndrome coronavirus (SARS-CoV), Crimean–Congo hemorrhagic fever virus (CCHFV), and Nipah virus (NiV) in platelets and plasma [29]. THERAFLEX^®^ UVC PR system also induced a mean log reduction greater than 3.5 against HEV [30].

The THERAFLEX^®^ UVC PR system is also effective in reducing transfusion-associated graft-versus-host disease (TA-GvHD) by inactivating leukocytes [31]. It differs from the INTERCEPT^®^ PR system and MIRASOL^®^ PR system as it does not require a photosensitizing agent. A German study involving 171 patients reported no significant side effects associated with the THERAFLEX^®^ UVC PR system [31]. However, this study was underpowered to detect side effects, and the primary efficacy outcome was not achieved, as PRT platelets were found to be less effective than standard platelets [9].

The THERAFLEX^®^ UVC PR system does carry some quality-related side effects, such as increased annexin V binding, lactate accumulation, and higher expression of CD41/61 [32]. Currently, the THERAFLEX^®^ PR system is the only PRT that provides time-to-treatment studies, which are crucial for determining the optimal time to start pathogen reduction treatment. The THERAFLEX^®^ PR system requires no photosensitizer, eliminating additional processing time for the removal of such chemicals. Consequently, it allows for the faster delivery of blood products and enhances safety by eliminating handling and transportation errors [33].

## 3. Advantages of PRT for Platelet Transfusions

Overall PRT has several effects which seem to be mainly positive. We have summarized and commented on most of those here below:

(i) PRT extends PLT shelf-life.
(ii) PRT and transfusion reactions.
(iii) PRT reduces Ta-GvHD.
(iv) PRT and TTI.
(v) PRT impact on coagulation proteins.
(vi) PRT harmonizes TTI risk between countries.
(vii) PRT does not affect the presence of pre-existing antibodies.
(viii) PRT and sepsis.

### 3.1. PRT Extends PLT Shelf-Life

The lifespan of platelets (PLT) is currently limited to 5 days. Therefore, any tool that could extend the shelf life of PLT is highly desirable for any blood transfusion service (BTS). The most widely adopted system for monitoring PLT contamination is BacT/ALERT^®^. This system is particularly prevalent in the UK and Ireland and helps monitor bacterial growth to prevent transfusion-transmitted infections (TTI). BacT/ALERT^®^ can identify contaminated PLT units at a very early stage. Recent studies have shown that BacT/ALERT^®^ can help extend PLT shelf life to 7 days by performing daily sampling for BacT/ALERT cultures and bacterial quantification [34]. Additionally, the INTERCEPT^®^ PR system has demonstrated the ability to preserve PLT quality over 7 days of storage [35]. The potential for extending PLT shelf life should be considered in any cost analysis of PRT implementation. A Spanish study indicated that PRT can help reduce the rate of PLT outdates [36].

### 3.2. PRT Reduces Transfusion Reactions

PRT has been associated with a reduction in PLT transfusion reactions [10,37]. The exact reason is unknown; however, this may simply be due to the inactivation of white blood cells and the consequent abrogation of cytokine production. Some authors have hypothesized a reduction in reactive stimuli by LPS, anti CD3+ and anti CD28 [38].

### 3.3. PRT Reduces Transfusion-Associated Graft versus Host Disease

The irradiation of blood products is used to prevent the development of transfusion-associated graft versus host disease (TA-GvHD). Indications for irradiated blood components include patients undergoing intrauterine transfusion, neonatal exchange transfusion, those with congenital cell-mediated immunodeficiencies, patients with Hodgkin lymphoma (for all transfusions during their lifetime), hematopoietic stem cell transplant recipients (both autologous and allogeneic), donors of allogeneic hematopoietic stem cells (in the week prior to and during stem cell harvest), CAR-T therapy recipients (7 days prior to collection and 3 months post-infusion), patients receiving purine analog therapy (e.g., fludarabine, cladribine, deoxycoformycin, bendamustine, clofarabine), those undergoing anti-thymocyte globulin (ATG) or alemtuzumab therapy for hematologic diseases, recipients of donations from biologic relatives, and recipients of donations selected on the basis of HLA matching. Early reports in large cohorts of patients in France and Switzerland have shown that PRT has strong efficacy for the prevention of TA-GvHD [39]. A study by Fast L and colleagues demonstrated that PRT is more effective than irradiation in the inactivation of T-cell lymphocytes and their proliferation [15]; irradiation does not fully impair CD69 expression [40]. Recently, Sim J and colleagues conducted a randomized prospective study to better evaluate the efficacy and safety of PRT. They studied a relatively large allogeneic transplant cohort (*n* = 30) where blood products were treated only with PRT and did not undergo any leukoreduction or irradiation [41]. This study was successful, as none of the patients developed TA-GvHD. This finding is particularly relevant within hematology, highlighting the potential of PRT to make blood product irradiation and leukoreduction redundant. Additionally, from an economic perspective, this could significantly reduce costs [42]. Liebermann and colleagues also commented on the prevention of CMV infection through leukoreduction, noting that PRT can effectively replace leukoreduction for CMV prevention [43].

### 3.4. PRT Reduces TTI 

The capacity of reducing the risk of TTI varies according to PRT type. It also differs if PLT are resuspended in plasma or in platelet additive solutions (PAS). The published data on INTERCEPT^®^ PR system showed a high efficacy against the following: (i) protozoa (Plasmodium falciparum, Trypanosoma cruzi, Leishmania mexicana, Leishmania major Jish, Babesia microti); (ii) Gram-negative bacteria (Escherichia coli, Serratia marcescens, Klebsiella pneumoniae, Pseudomonas aeruginosa, Salmonella choleraesuis, Yersinia enterocolitica, Enterobacter cloacae, Anaplasma phagocytophilum); (iii) Gram-positive bacteria (Staphylococcus epidermis, Staphylococcus aureus, Streptococcus pyogenes, Listeria monocytogenes, Corynebacterium minutissimum, Bacillus cereus (vegetative), Bifidobacterium adolescentis, Propionibacterium acnes, Lactobacillus species, Clostridium perfringens (vegetative form), Spirochete Bacteria such as Treponema pallidum and Borrelia burgdorferi); (iv) enveloped viruses (HIV-1, HBV (strain MS-2), HCV (strain Hutchinson), HTLV-I, HTLV-II, CMV, Bovine Viral Diarrhea Virus, Duck Hepatitis B Virus, Pseudorabies virus, West Nile Virus (WNV), SARS-CoV2, Chikungunya virus, Influenza A H5N1 virus (Avian Influenza, Zika, Dengue, Yellow Fever); (v) non-enveloped viruses (Bluetongue Virus type 11, Calicivirus, Human Adenovirus-5, Parvovirus B19) [44,45]. The technical data sheet available on MIRASOL^®^ PR system is not so informative, but clearly states that even this PRT is efficacious against enveloped and non-enveloped viruses, gram-negative and gram-positive bacteria, and parasites [46].

In the case of the THERAFLEX^®^ UVC PR system, activity against the following bacteria is reported: Enterobacter cloacae, Escherichia coli, Klebsiella Pneumoniae, Morganella morganii, Proteus mirabilis, Pseudomonas fluorescens, Serratia marcescens, Staphylococcus aureus, Staphylococcus epidermidis, Streptococcus bovis, Streptococcus dysgalactiae, Streptococcus pyogenes, Listeria monocytogenes, Acinetobacter baumannii, Streptococcus agalactiae, Streptococcus pneumoniae, Bacillus cereus, Bacillus thuringiensis, Propionibacterium acnes [47]. Also, activity against parasites such as Babesia divergens [48] and African mosquito-borne flaviviruses such as WNV and Usutu virus (USUV) have been shown [49].

The use of PRT is quite promising. At present, there are several reports on the use of PRT. For example, Pitman reported on 1,221,031 platelet transfusions in France over 2010–2020 [37]. Escolar and colleagues reported that >875,000 transfusion treated with INTERCEPT^®^ and >700,000 treated with MIRASOL^®^ have been administered so far, and no case of TTI or sepsis was reported [26].

### 3.5. PRT Impact on Plasma Coagulation Proteins

Plasma products treated with PRT will have reduced levels of key coagulation proteins. Statistically significant reductions in FII, FV, FVIII, FIX, and FXI have been shown [50]. However, this may not necessarily impair the hemostatic function of the plasma product. For example, a thrombin generation (TG) study conducted on plasma treated with INTERCEPT^®^ PR system showed TG levels similar to those of untreated plasma, suggesting that hemostatic function is preserved [51].

Studies have shown that plasma treated with solvent/detergent or with methylene blue (MB) can affect Protein-S levels [52,53,54]. This is relevant because Protein-S is a co-factor of Protein-C and is essential for its conversion into activated Protein-C (APC). APC balances the actions of FVa and FVIIIa [55]. MB-treated plasma best preserves FV and FXI levels compared to the INTERCEPT^®^ PR system [56]. This information is relevant because often platelets (PLT) are resuspended in plasma [57].

## 4. Criticisms and Limitations of PRT

The following points were identified as criticisms and limitations of PRT:

(i) Bleeding risk.
(ii) PLT increment count.
(iii) Biochemical changes.
(iv) Mitochondrial DNA inactivation.
(v) PRT alters microRNA.
(vi) Pathogens not impacted by PRT.
(vii) Costs.

### 4.1. Bleeding Risk and PLT Count Increment 

The possibility that PRT may affect platelet quality and contribute to a higher bleeding risk has been a concern for several investigators and continues to be so for some. An exhaustive Cochrane review on the risk of bleeding associated with PRT was carried out in 2017 [58]. More recently, a systematic review of all published randomized controlled trials was published by the Italian group of Pati I in 2022. In this study, Pati I and colleagues reported no difference in clinically significant bleeding between the INTERCEPT^®^ PR system and the MIRASOL^®^ PR system. However, a higher number of bleeding events was observed in the PRT groups [59].

Among these studies, the most concerning is the one conducted by the Dutch group of Kerkhoffs JLH and colleagues [14]. In this study, a reduced 1 h corrected count increment (CCI) was documented using the INTERCEPT^®^ PR system. Moreover, an increased risk of bleeding was also documented. Interestingly, patients with hematological malignancies such as AML, ALL, and LNH were included. This is relevant since these patients have very low PLT counts and are quite prone to bleeding.

Similarly, the Italian Platelet Technology Assessment Study (IPTAS) showed that the absolute risk difference in WHO Grade ≥ 2 bleeding for the INTERCEPT^®^ PR system was 6.1% (with an upper one-sided 97.5% confidence limit of 19.2%), while the absolute risk difference for the MIRASOL^®^ PR system was better at 4.1% (with an upper one-sided 97.5% confidence limit of 18.4%) [60]. However, in neither case was the absolute risk difference statistically significant. In both trials, post-transfusion platelet count increments were significantly lower in treated versus control patients [60].

A randomized French study (EFFIPAP) did not show that PLTs treated with PRT (INTERCEPT^®^ PR system) and resuspended in PAS were associated with any higher rate of WHO Grade ≥ 2 bleeding. However, a 12.5% difference margin was considered for this non-inferiority study [61].

Osselaer JC and colleagues studied a large cohort of patients (*n* = 651) treated with 5106 blood components transfused with the INTERCEPT^®^ PR system. The authors reported no changes in the number of PLT doses administered with and without PRT implementation [62]. Cazenave JP and colleagues also reported similar findings. Interestingly, these authors also found a reduced number of transfusion reactions associated with PRT using the INTERCEPT^®^ PR system [63]. More recently, an Austrian study reported a similar consumption of blood products before and after PRT implementation (INTERCEPT^®^ PR system) over 21 months of follow-up. A similar finding was also reported by Infanti L and colleagues, in a study that also showed the possibility of extending PLT shelf life to 7 days [64].

This suggests that no significant increase in bleeding was seen with PRT implementation [65]. The same group of authors also studied a large cohort of patients treated with massive transfusion [66]. Interestingly, no increased bleeding risk was seen after PRT implementation. Additionally, both the SPRINT [67] and the EFFIPAP [61] studies enrolled patients with severe hematological conditions and low PLT counts, suggesting that these “fragile” patients are not adversely affected when PRT is implemented.

### 4.2. PLT Increment Count 

Several reports have suggested that PRT-treated PLT lead to an inferior post-transfusion PLT count compared to that obtained when PLT without any PRT treatment are transfused [62]. To analyze this aspect, the concept of a corrected count increment (CCI) has been introduced. The CCI was initially developed with the intent to facilitate physicians dealing with differential diagnosis of idiopathic thrombocytopenic purpura (ITP) and is currently used for analyzing platelet refractoriness. This index directly correlates to the PLT dose transfused. The CCI is based on a small calculation on PLT numbers based on a post-transfusion specimen collected 1 h after transfusion (CCI_1hour_). This index also considers the patient’s body surface area (BSA). Usually, after a transfusion of a standard unit of PLT in a patient with not-active bleeding, the PLT count shows an increment between 5 and 10.000/µL.

$$\mathrm{CCI}=\frac{[\left(\mathrm{post}-\mathrm{transfusion}\;\mathrm{PLT}\;\mathrm{count}\;1\;h\;\mathrm{after}\;\mathrm{transfusion}/\mu L\right)-\left(\mathrm{pretransfusion}\;\mathrm{PLT}\;\mathrm{count}/\mu L\right)]\;\times \;\mathrm{BSA}}{\left(\mathrm{number}\;\mathrm{of}\;\mathrm{PLT}\;\mathrm{transfused}\right)\times {\;10}^{11}/\mu L}$$

If the CCI does not show an increment, further investigations would be required to identify the cause (e.g., immunization to HLA- or HPA-antigens, ITP, sepsis, etc.). A Canadian simulation study reported on the possible risk of wastages and PLT shortages caused by PRT implementation. The authors described a reduced CCI when the INTERCEPT^®^ PR system was used. This would have led to prescribing more products, and enhanced patient’s exposure to more blood donors. The authors concluded that this would impact on supply chain and potentially increase the risk of TTI because of larger exposure to blood products [68]. Different results were shown by the SPRINT randomized study. In particular, the CCIs at 1 h and at 24 h post-transfusion were lower for patients transfused with PRT-treated PLT. Also, the transfusion interval was shorter leading overall to a higher prescription of PLT; however, no differences in clinical bleeding were seen [69]. However, other studies have not confirmed these data and have not reported any increased PLT consumption for the INTERCEPT^®^ PR system [62,63,64,65,66].

### 4.3. Biochemical Changes 

Malvaux N et al. reported on changes in biochemical markers in PLT concentrate treated with either the MIRASOL^®^ PR system or the INTERCEPT^®^ PR system. A higher production of lactate, along with a greater reduction in glucose, was observed for MIRASOL^®^. Swirling at day 5 was also worse when PLT were treated with the MIRASOL^®^ PR system, suggesting that the INTERCEPT^®^ PR system should be preferred [23]. Conversely, PLT treated with the INTERCEPT^®^ PR system preserved swirling until day 7 [23,35]. However, some biochemical studies have shown problems even when the INTERCEPT^®^ PR system is used. Specifically, Stivala S and colleagues reported that PLT treated with the INTERCEPT^®^ PR system exhibited reduced function in response to activation agonists (i.e., collagen, thrombin, and von Willebrand Factor). The same study showed increased PLT apoptosis through Bak upregulation and a caspase-dependent pathway, enhancing PLT clearance from the circulation, with obvious clinical implications [70].

### 4.4. Mitochondrial DNA Inactivation

Following ultraviolet (UV) light-based pathogen inactivation, mitochondrial DNA (mitDNA) damage can occur. Specific PCR multiplex assays have been developed for testing mitDNA. These assays have demonstrated that pathogen reduction technology is responsible for this damage [71,72]. Furthermore, Bakkour and colleagues showed that mitDNA damage is primarily UV-dependent and not related to the presence of amotosalen [73].

### 4.5. PRT Alters microRNA 

Diallo I and his colleagues were the first to report alterations in microRNAs contained in platelet-derived microparticles (PMP) [74]. The authors suggested that pathogen reduction technology might influence microRNA loading into PMP, which may indirectly affect PMP bioactivity. Alterations of microRNAs (miRNAs) stored within PMP were observed with the INTERCEPT^®^ PR system. No such alterations were seen with the MIRASOL^®^ PR system. The authors attributed these alterations to the presence of the additive storage solution (amotosalen) used with INTERCEPT^®^. Arnason and colleagues reported that the INTERCEPT^®^ PR system affects only the expression of miRNA-96-5p, showing downregulation of this particular miRNA out of the 25 miRNAs analyzed [75].

### 4.6. Pathogens Not Impacted by PRT 

PRT can significantly contribute to reducing the pathogen load associated with transfusion, but no type of PRT ensures the full sterility of blood products. Additionally, PRT is clearly ineffective against prions, spores, and some viruses without a lipid envelope (e.g., Hepatitis A). However, the MIRASOL^®^ PR system and the THERAFLEX^®^ UVC PR system have been shown to inhibit HEV [20].

### 4.7. PRT High Costs

The cost analysis of implementing PRT varies between studies and in different geographical, epidemiological and economical settings. Several factors affect this cost, including the type of PRT procedure, shorter intervals of sustained transfusion, extended shelf-life from 5 to 7 days, the elimination of test procedures (such as CMV testing and bacterial culture), the elimination of gamma irradiation.

Cicchetti A and colleagues conducted a cost analysis on PRT in Italian patients, considering over 50,000 transfused patients in one year. They predicted an overall cost increase of 30% [6]. Additionally, the same authors suggested that while PRT can significantly reduce the risk of transfusion-transmitted infections (TTI), it does not guarantee protection against all TTIs. Furthermore, they indicated that PRT is not applicable to all blood products (e.g., red cells) and may cause alterations in product quality [6]. 

In a constructive criticism, Farrugia emphasized that although current PRT is not perfect, previous regulatory authorities, such as the US Blood Product Advisory Committee, investigating blood inquiries related to HIV transmission, have clearly stated that “partial solutions should be welcome and where possible implemented by BTS” [76,77]. We concur with this statement. Our perspective is that ensuring and improving patient safety should always remain a priority for any BTS.

CF Bell and colleagues, in an earlier cost analysis, also noted that introducing PRT would render bacterial testing redundant, leading to indirect cost savings [78]. The cost saving associated with the prevention of a potentially emerging virus is very significant when considered in economic studies.

Most studies on cost analysis have been conducted on the INTERCEPT^®^ PR system only. In this respect, a study sponsored by INTERCEPT showed that by adopting PRT the incremental cost effectiveness ratio (ICER) of PLT is certainly higher; however, this may be justified if new emergent pathogens arise with a 10-fold risk of transmission with transfusion [79]. Gregoire Y and colleagues from Québec showed an ICER on quality-adjusted life years (QALY) equal to USD 8.1 million/QALY [80]. However, in the presence of a new blood-borne pathogen the ICER was reduced to USD 123,063. Rosskopf K and his colleagues reported on the advantages of not having gamma irradiation and bacterial screening in place. The analysis concluded that implementing PRT added an additional cost of +7.5% [81]. Mc Cullough and colleagues reported on significant cost saving for each PLT unit treated with PRT, if standard microbiological testing were removed. Furthermore, the same authors stressed that a shelf life extension to 7 days would contribute significantly to cost reduction [12].

## 5. Future Perspectives

### Potential Improvements and Advancements in PRT

The prevention of TTI cannot prescind from implementing PRT or similar systems for red cell concentrate (RCC) and plasma products. Recently, a randomized trial called ReCePI was conducted on the INTERCEPT^®^ PR system for RCC in patients undergoing cardiovascular surgery. The trial demonstrated non-inferiority for the INTERCEPT^®^ PR system-treated RCC compared to conventional RCC as measured by the incidence of acute kidney injury (AKI).

Similarly, studies on fibrinogen compounds like cryoprecipitate are currently ongoing [82].

## 6. Analysis of Arguments for and against PRT 

A critical review of previous randomized control trials (RCT) did not identify relevant clinical complications when PRT was implemented. For example, the worrisome higher bleeding (>WHO grade 2) or lower CCI did not really emerge, even if data have shown that PRT may impact on the post-transfusion PLT count. From the perspective of improving blood safety, there is no clear reason why PRT should not be further implemented. The economic cost associated with PRT implementation may have been an obvious obstacle. However, considering the benefits of PRT (reduced risk of TTI, better TA-GvHD prevention and no need of irradiation), it is not clear to these authors why PRT has not been widely adopted. Perhaps, the large number of RCT designed to prove that PRT does not impact on product quality (reduced CCI count, or increased bleedings), may have contributed to creating the perception that PRT is unsafe. Interestingly, those centers that have already adopted PRT have not moved away from this practice. This indirectly validates PRT. However, the wait and watch approach adopted by other BTS indicates that a large resistance towards implementing PRT still exists.

## 7. Conclusions

### 7.1. Summary of Key Points

TTI in transfusion medicine have always been a challenge, particularly because new emerging disease will always be present, even in the future. The recent COVID-19 pandemic is a good example of this. For this reason, PRT in transfusion medicine offers the possibility to reduce and control future events caused by unknown pathogens. The relationship between zoonosis and TTI (West Nile Virus, Zika, MERS, SARS, Malaria, Chikungunya virus, etc.) is large and well reported in the literature. The possibility to implement blood safety by using UV lights with or without intercalating agents able to block DNA and RNA replication signaling, is likely to become a cornerstone of transfusion medicine. Moreover, the introduction of this technology will even potentiate the safety of the existing PCR screening practice, ensuring that any false negative samples that are potentially infective will still undergo inactivation. This transfusion concept has also been recently debated by Farrugia in his description of a “safety tripod” made by (1) appropriate donor selection, (2) blood screening with molecular technology, and (3) the adoption of viral inactivation processes [77].

### 7.2. Personal Recommendation and Final Remarks

An unquestionable advantage of introducing PRT is the potential to extend the shelf life of platelets (PLTs), which would offer significant inventory management benefits by making more products available for longer periods of time. Although implementing PRT in low-income countries, such as those in sub-Saharan Africa where the risk of transfusion-transmitted infections (TTIs) is high, could theoretically be beneficial, it remains impractical and unrealistic due to the high costs involved [18]. Even in resource-rich countries, however, the widespread adoption of Pathogen Reduction Technology (PRT) encounters resistance, often due to objective concerns such as potential damage to blood products, adverse effects like alloimmunization, logistical challenges, and significant costs. While PRT may offer substantial benefits in specific epidemiological scenarios, it is essential for each national transfusion authority to define the most appropriate strategy based on an evidence-based approach. What is needed now is a shared risk assessment methodology across different countries to support decision-making, ensuring that the process is as objective and reproducible as possible, thereby guaranteeing all patients access to care that is balanced in terms of both efficacy and safety.

## Figures and Tables

**Table 1:** Summary of key characteristics of the three types of PRT.

	Intercept PR System	Mirasol PR System	Theraflex UVC PR System
UV Type	UVA	UVB	UVC
Mechanism of action	Following exposure to UVA, an amotosalen compound will crosslink to DNA and RNA chains.	Riboflavin (Vitamin B2) associates with nucleic acids and mediates an oxygen-independent electron transfer process leading to the modification of DNA/RNA upon exposure to UV light.	
Adsorption device	The residual amotosalen is removed using a compound adsorption device (CAD).	Not needed.	Not needed.
Toxicity	No toxicologically relevant effects. The safety margins of amotosalen alone in toxicity studies are >1000.	No toxicity.	No toxicity.
Advantages	Excellent microbiological spectrum.	Good microbiological spectrum.	Good microbiological spectrum. Cost contained.
Disadvantages	High cost. Possibility of some saving by treating double dose.	High cost.	Does not protect against a large spectrum of microbiological agents. Large studies are lacking.

## Abbreviations

AKI	acute kidney injury
ALL	acute lymphoblastic leukaemia
AML	acute myeloid leukaemia
APC	activated Protein C
ATG	anti-thymocyte globulin
BSA	body surface area
B-SCEP	Blood Supply Contingency and Emergency Plan
BTS	Blood Transfusion Service
CAD	compound adsorption device
CCI	corrected count increment
CCHFV	Crimean-Congo hemorrhagic fever virus
CMV	cytomegalovirus
EDQM	European Directorate for the Quality of Medicines
FFP	fresh frozen plasma
GvHD	Graft versus Host Disease
HEV	hepatitis E virus
HIV	human immunodeficiency virus
ICER	incremental cost effectiveness ratio
IPTAS	Italian Platelet Technology Assessment Study
ITP	idiopathic thrombocytopenic purpura
LNH	lymphoma non-Hodgkin
LRF	Log Reduction Factor
MB	methylene blue
mitDNA	mitochondrial DNA
NAT	nucleic acid testing
NiV	Nipah virus
PAS	platelet additive solution
PCT	photochemically treated
PLTs	platelets
PMP	platelet-derived microparticles
PR	pathogen reduction
PRT	Pathogen Reduction Technology
QALY	quality-adjusted life years
RCC	red cell concentrate
RCT	randomized control trials
RSV	respiratory syncytial virus
SARS-CoV	severe acute respiratory syndrome coronavirus
SD	solvent detergent
TA-GvHD	transfusion-associated graft-versus-host disease
TG	Thrombin generation
TTI	Transfusion-transmitted Infections
USUV	Usutu virus
UV	ultraviolet
vCJD	variant Jacob Creutzfeldt Disease
WNV	West Nile virus
